# Pipeline Embolization Stent for the Treatment of Giant Supraclinoid Aneurysms: A Case Series

**DOI:** 10.7759/cureus.23674

**Published:** 2022-03-31

**Authors:** Michael Fana, Owais Alsrouji, Mohammed Rehman

**Affiliations:** 1 Neurology, Henry Ford Health System, Detroit, USA; 2 Neurological Surgery, Henry Ford Health System, Detroit, USA

**Keywords:** interventional radiology-guided embolization, multiple cranial nerve palsy, endovascular stenting, cavernous carotid artery aneurysm, endovascular aneurysm repair

## Abstract

Cerebrovascular aneurysms of the supraclinoid region are a technical challenge and can be particularly difficult to treat when greater than 25 mm in diameter. Such giant aneurysms can be approached with various skull-based and endovascular surgical techniques, and the advent of the Pipeline embolization stent presents a new treatment modality. Previously used for the treatment of small aneurysms, the Pipeline embolization device (PED) is a flow diverter device that has more recently been investigated in its use for the treatment of giant aneurysms with few studies to date published about its procedural outcomes. Here, we highlight the case of three patients (two elderly and one middle-aged) presenting symptomatically with giant supraclinoid aneurysms of the cavernous internal carotid artery (ICA) and posterior communicating artery treated with the Pipeline stent and monitored on follow-up visits. We further review the most current case reports and the two clinical trials to date investigating the utility of the Pipeline stent in the treatment of large and giant cerebral aneurysms, highlighting the emerging evidence of its efficacy and long-term patient outcomes. We report successful resolution of symptoms and radiographic evidence of aneurysm size reduction on all patient follow-ups and suggest the Pipeline embolization device as a novel technique that can be utilized for the treatment of giant cerebrovascular aneurysms with emerging evidence of immediate and long-term success.

## Introduction

Cerebrovascular aneurysms pose a challenge in treatment and symptom management to date. Traditionally, aneurysm clipping by way of craniotomy demonstrates a long-standing history of excellent treatment and prevention of recurrence approach. However, this is not without significant risk posed to patients, particularly when involving skull-based aneurysms in anatomically challenging locations such as the cavernous sinus. At the margin of the supraclinoid segment where internal carotid arteries (ICA) enter the subarachnoid space, aneurysms in this area can be technically challenging to access. With the advent of endovascular neurosurgery, treatment options such as endovascular coiling, onyx embolization, and ICA parent artery occlusion now exist as alternative approaches for treatment. Flow diverter devices including the Pipeline embolization device (PED) (Covidien, Mansfield, MA, USA) and SILK (Balt Extrusion, Montmorency, France) are new innovations providing the option for implanting a thrombogenic stent to establish flow diversion away from the aneurysm while allowing for the formation of a neointimal layer of the inner blood vessel lumen. The PED is a flexible stent composed of 25% platinum-tungsten and 75% cobalt-chromium-nickel alloy with a stent porosity of 70%, providing the flexibility of vessel conformity and appropriate metal coverage of the vessel [1]. In the ongoing clinical trial, PREMIER, prospective results to date indicate a 76.8% success in the complete occlusion of the aneurysm on one-year follow-up angiography of 141 patients with a major morbidity and mortality rate of 2.1% [2].

PED use for the treatment of giant and large aneurysms was FDA-approved in 2011 and has since been used for the treatment of aneurysms of various sizes, types, and locations, including off-label uses for dissecting aneurysms, carotid-cavernous fistulas, pseudoaneurysms, and ruptured aneurysms [3]. Its use for giant aneurysms, defined as aneurysms with a diameter greater than 25 mm, is currently an ongoing investigation for postoperative outcomes and recurrence rates with few reports in the literature. Here, we present a series of three patients presenting with giant intracerebral aneurysms in the supraclinoid ICA with symptomatic presentations. We report success in the placement of a Pipeline embolization device in each case with resolution of presenting symptoms on follow-up examination.

## Case presentation

Case 1

**Figure 1:**
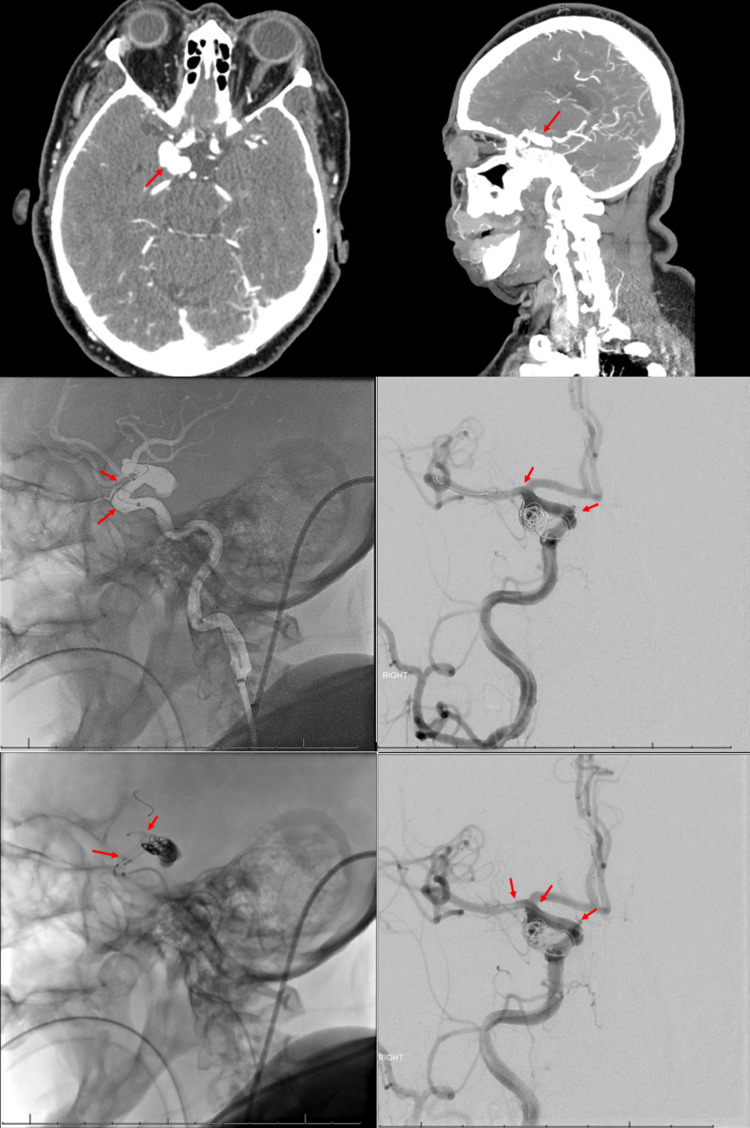
PED placement of right posterior communicating artery aneurysm Top row: CT angiography in axial and sagittal views, respectively, demonstrating an 18 mm multi-lobular, irregular, broad-based right posterior communicating artery aneurysm extending to the right carotid terminus (arrows). Middle row:Digital subtraction angiography (DSA) demonstrating guiding catheter placement of a triple coaxial system with a Navien catheter and Phenom catheter placed in the middle cerebral artery M1/M2 segment (red arrows). An SL-10 microcatheter is placed within the posterior communicating artery aneurysm neck, and a 9 × 30 XL target 360 soft coil was partially deployed within the aneurysm. Bottom row: Next, a 4.5 × 20 mm Pipeline embolization device was successfully deployed across the neck of the right posterior communicating artery aneurysm (red arrows) with a final angiogram demonstrating successful coiling and PED placement in the right posterior communicating artery aneurysm.

Our first patient is a 69-year-old patient with a medical history of hyperlipidemia and hypertension and a 50-pack year tobacco smoking history presenting with left-sided facial hemiparesis and left-sided upper and lower extremity hemiparesis. CT angiography demonstrated an 18 mm multi-lobular, irregular, broad-based right posterior communicating artery aneurysm extending to the right carotid terminus. The patient was started on aspirin 81 mg and atorvastatin 80 mg without the need for acute intervention. Stroke etiology was determined to be secondary to small vessel disease, and the patient was discharged with an NIHSS of 2 and mRS of 3. The patient was instructed to return for endovascular therapy at a three-month follow-up. The patient underwent Pipeline stent placement with coiling of the 25 × 14 × 7 mm supraclinoid aneurysm without any intra-procedural complications (Figure 1).

The patient was discharged on dual antiplatelet therapy with follow-up in six months. On follow-up visits at four and six months, the patient endorsed complete resolution of initial symptoms without any new focal neurological deficits (Table 1).

Case 2

**Figure 2:**
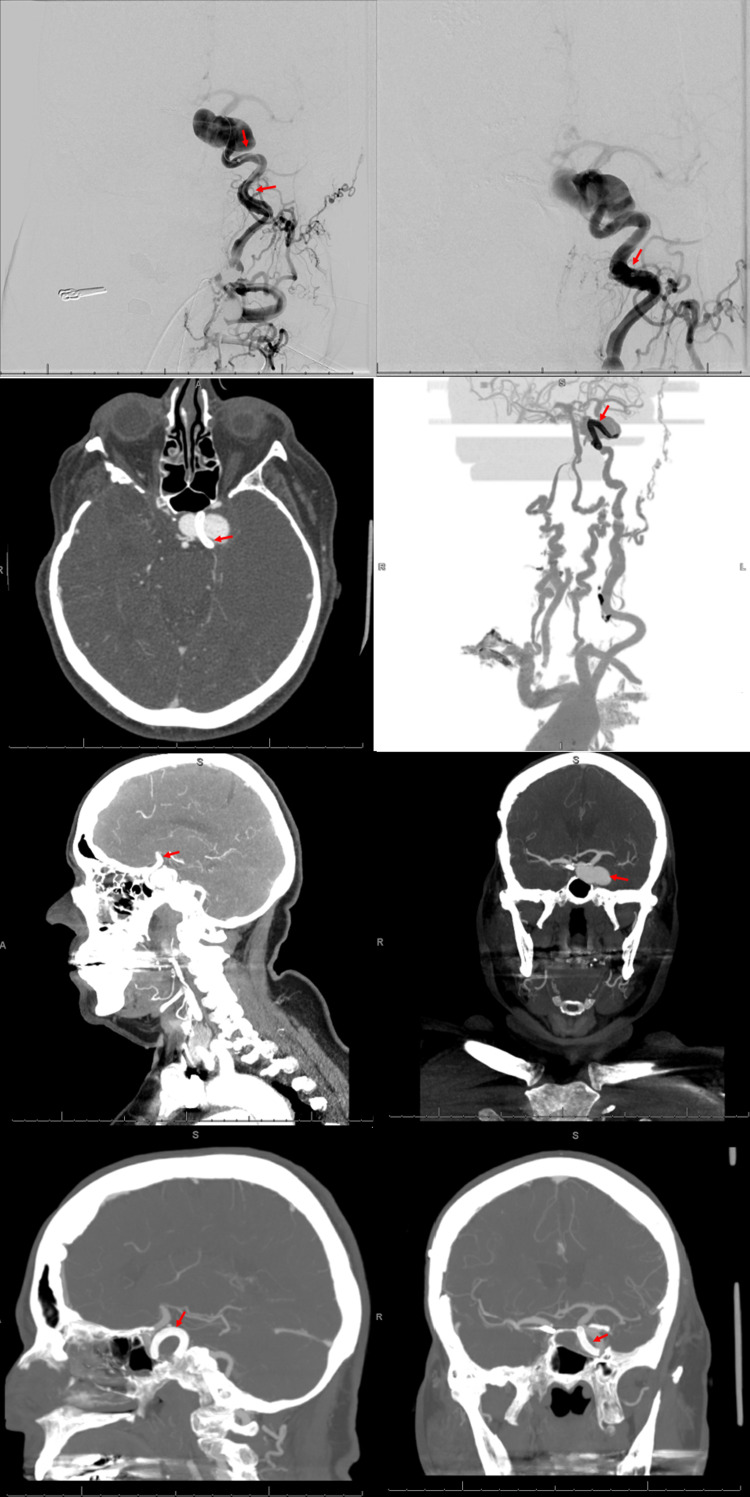
PED placement of right cavernous ICA aneurysm Top row, left: Digital subtraction angiography (DSA) demonstrating the use of a quad axial technique with the long sheath, flow gate guide catheter, and phenom plus in the distal cervical internal carotid artery. The aneurysm was then approached using a Synchro microguidewire. The ophthalmic segment and supraclinoid segment of the internal carotid artery were catheterized. The Asahi 18 microguidewire was used to provide support for this catheter to be placed near the M1 carotid terminus as indicated by the red arrows. Seen as well is the previous surgical clip placement of the right ICA. Top row, right: A 4.25 × 30 mm PED was deployed successfully across the neck of the aneurysm with the tail end of the stent noted by the red arrow. Second row: Postoperative follow-up axial non-contrast CT of the head and with reconstruction demonstrating PED placement across the neck of the supraclinoid aneurysm (red arrows). Third row: Initial preoperative CT of the head with contrast in sagittal and coronal views demonstrating an approximately 27 × 15 × 15 mm cavernous ICA aneurysm without evidence of occlusion or stenosis (red arrows). Bottom row: Six-month follow-up CT angiography in sagittal and coronal views demonstrating absent filling of left cavernous ICA aneurysm with patent stent placement indicated by the red arrows.

Our next patient is a 77-year-old patient with a medical history of a previously clipped non-ruptured right cavernous ICA aneurysm, hypertension, and hyperlipidemia presenting with left eye proptosis, retro-orbital pain, and unilateral left-sided lateral rectus palsy. Non-contrast CT of the head and neck demonstrated a large, left-sided aneurysm in the cavernous internal carotid artery confirmed on CT angiography as a 28 × 20 × 18 mm giant aneurysm. The patient was discharged without acute intervention with aspirin 325 mg and clopidogrel 75 mg with follow-up for endovascular neurosurgical intervention. The patient later underwent successful flow diversion with placement of a Pipeline stent across the neck of the aneurysm (Figure 2).

Intra-procedural complication included a distal thromboembolic event in the left MCA and ACA territory manifesting as right upper and lower extremity hemiparesis, mild dysarthria, and partial unilateral facial paralysis with a new NIHSS of 8. The patient received intra-arterial integrelin and postoperatively was discharged on dual antiplatelet therapy. At one-month and 10-month follow-ups, the patient demonstrated a significant recovery in extremity strength, facial mobility, and vision with only mild residual unilateral hemiparesis (Table 1). At the six-month follow-up, CT angiography of the head demonstrated a patent cavernous carotid stent placement without opacification of the aneurysm (Figure 2).

Case 3

**Figure 3:**
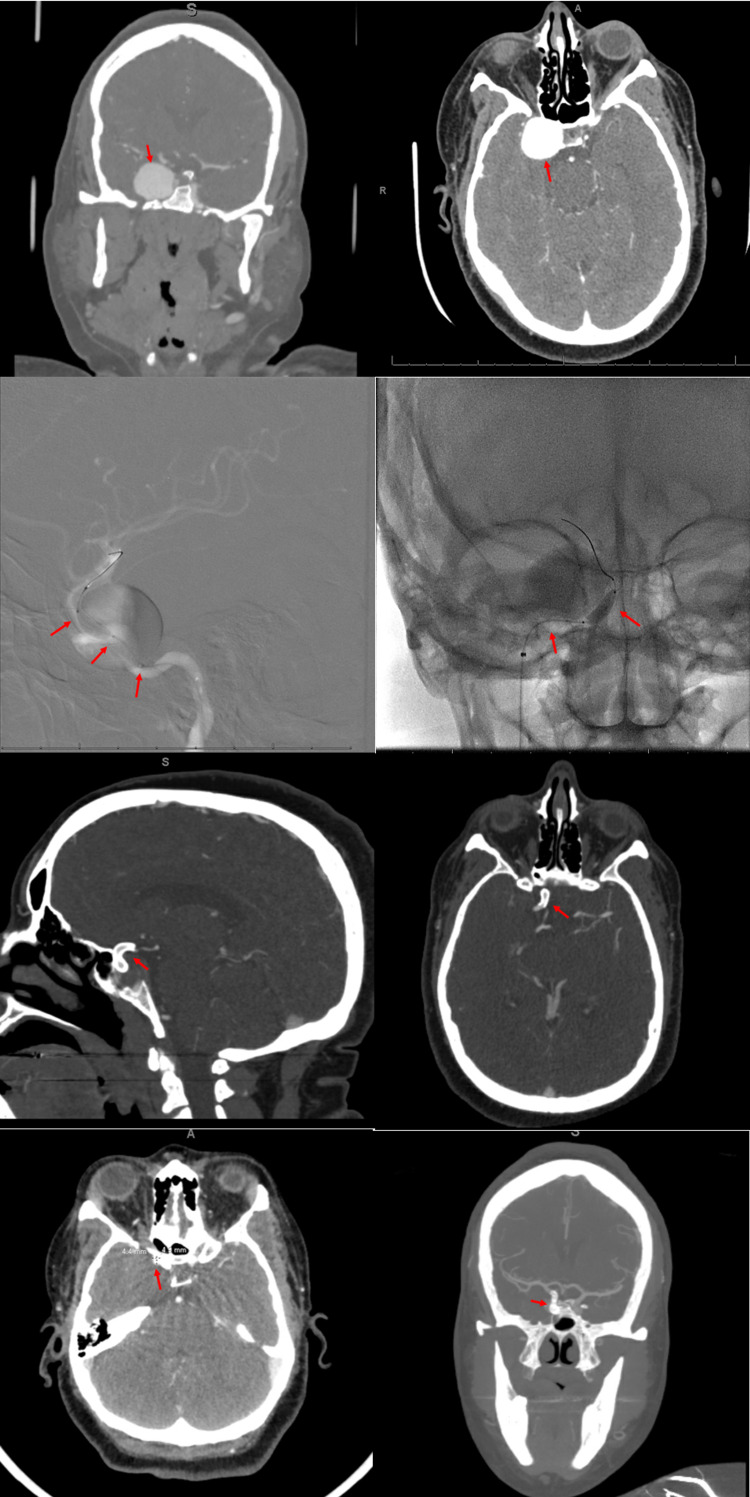
PED placement of right cavernous ICA aneurysm Top row: Coronal and axial CT angiography of the head demonstrating a right cavernous ICA saccular aneurysm measuring approximately 26 × 26 × 23 mm without intracranial occlusion or stenosis (red arrows). Second row: A triple coaxial system with a phenom plus intermediate catheter, and phenom 27 microcatheter was used and placed in the proximal middle cerebral artery. Subsequently, a 5 × 20 mm Pipeline embolization device was placed across the neck of the aneurysm (red arrows). Stent placement was angioplastied with a Scepter C angioplasty balloon for initial incomplete stent apposition at the proximal end. The stent was successfully angioplastied with repeat imaging shown here without evidence of occlusion and stasis of the aneurysm. Third row: Postoperative sagittal and axial non-contrast CT of the head demonstrating right internal carotid artery stent along the cavernous to supraclinoid ICA (red arrows). Fourth row: One-month follow-up axial and coronal CT angiography of the head demonstrating an approximate 5 × 5 mm area of opacity across the stent body at the supraclinoid segment projecting laterally as indicated by the red arrow, likely from incomplete occlusion of the saccular aneurysm.

Our third patient is a 45-year-old patient with a medical history of refractory hypertension and a 20-pack year tobacco smoking history presenting with severe headaches, unilateral right-sided eye proptosis, pupillary dilation, and lateral rectus palsy for the past two months. CT angiography demonstrated a 21 × 20 × 26 mm saccular aneurysm of the right supraclinoid internal carotid artery. The patient was loaded with aspirin 325 mg and clopidogrel 600 mg prior to endovascular flow diversion of the aneurysm. They underwent Pipeline stent placement across the neck of the aneurysm without coiling and without any intraoperative or postoperative complications. The patient was discharged on dual antiplatelet therapy for six months (Figure 3).

On follow-up appointment at one month, the patient endorsed continued unilateral right-sided headaches, retro-orbital pain, and neck pain. CT angiography at the time demonstrated patency of the stent with a 5 × 5 mm area of enhancement adjacent to the supraclinoid internal carotid artery, likely due to a small residual aneurysm (Figure 3). The patient’s headaches were managed with venlafaxine and magnesium oxide, and on the three-month follow-up, the patient endorsed complete resolution of headaches with only residual mild lateral rectus palsy (Table 1).

**Table 1:** Key features of patients presented in this case series, including presenting symptoms, risk factors, medical and surgical interventions utilized, descriptive features of the aneurysm, results and intraoperative complications, and radiographic follow-up results

Age	Presenting Symptoms	Risk Factors	Medical Interventions	Surgical Interventions	Aneurysm Size and Location	Follow-Up Results	Follow-Up Imaging Results	Intraoperative Complications
69	Unilateral facial palsy, unilateral upper and lower extremity hemiparesis	Hyperlipidemia, hypertension, tobacco smoking (50-pack year), NIDDM, obesity (BMI > 30)	Aspirin, atorvastatin, lisinopril, clopidogrel	(1) 4.5 × 20 mm Pipeline stent, (1) 9 × 30 XL 360 soft coil	25 × 14 × 7 mm supraclinoid PComm	Complete symptom resolution (three months)	None	None
77	Unilateral proptosis, retro-orbital pain, unilateral lateral rectus palsy	Hyperlipidemia, hypertension	Aspirin, clopidogrel, hydrochlorothiazide (HCTZ), lisinopril, lovastatin	(1) 4.25 × 30 mm Pipeline stent	28 × 18 × 20 mm cavernous ICA	Complete symptom resolution (one and 10 months)	Patent cavernous carotid stent placement without aneurysm filling (six-month CTA)	Distal thromboembolism in the left MCA and left ACA territories with NIHSS of 8
45	Unilateral proptosis, pupil dilatation, unilateral lateral rectus palsy, intractable headaches	Hypertension (uncontrolled), tobacco smoking (20-pack year)	Aspirin, clopidogrel, lisinopril, metoprolol, amlodipine, hydralazine, Lasix	(1) 5 × 20 mm Pipeline stent, (1) 4 × 10 mm angioplasty balloon	21 × 20 × 26 mm cavernous ICA	Partial symptom resolution - residual mild lateral rectus palsy (three months)	Patent stent with a 5 × 5 mm area of enhancement adjacent to the supraclinoid internal carotid artery (one-month CTA)	None

## Discussion

Few studies to date have investigated the PED for its utility in treating large and giant aneurysms. A thorough review of the literature on PubMed with inclusion criteria of “Pipeline Embolization Device,” “Pipeline stent,” “Flow diverter device,” and “Giant cerebral aneurysms” and exclusion criteria of non-cerebral aneurysms and aneurysms smaller than 25 mm in diameter yielded five case reports, one retrospective chart review, and two clinical trials. In one study by Briganti et al. (2014), 35 patients were enrolled, of which 30 underwent PED placement and five with SILK. Only one patient had a giant aneurysm, and six had large aneurysms. Similar to our patients, the majority were supraclinoid ICA aneurysms, and complete occlusion was attained in 35 of the 38 total aneurysms, with the remaining three achieving partial occlusion within six months [1]. In another study by Carneiro et al. (2014), eight patients were treated with flow diverter devices for giant aneurysms, and likewise, four were cavernous ICA aneurysms. Five patients were treated with PED, while three were treated with SILK. The results at 6- to 48-month follow-up were variable with findings of two of the cavernous aneurysms decreasing in size, one remaining unchanged, and one increasing in size [4]. The use of a flow diverter device has also been used in conjunction with coiling in literature in a female patient with a ruptured giant cavernous segment ICA aneurysm. In a six-month follow-up digital subtraction angiography (DSA), only minimal residual fistulous flow was found draining consistent with rapid recovery. One-and two-year follow-up DSA demonstrated subsequent obliteration of the fistula with stable occlusion of the aneurysm, suggesting that the concurrent use of both stenting and coiling may provide more appropriate long-term aneurysm management [5].

PEDs have also been demonstrated to be used effectively in patients presenting with a giant fusiform aneurysm in the cavernous ICA, with aneurysmal shrinkage at two-year follow-up, indicating its utility in such aneurysms [6]. In cases involving cavernous ICA aneurysms, cranial nerve neuropathies may be a commonplace finding, including facial hemiparesis, oculomotor dysfunction, visual deficits, and facial hemisensory deficits. In other rarer instances, pituitary dysfunction as a consequence of a giant cavernous ICA aneurysm can occur in 0.2% of patients, with good resolution of symptoms utilizing the PED [7]. Nevertheless, several limitations exist in the use of PEDs for the treatment of aneurysms of various sizes, including those of the distal and posterior circulation. Few studies to date have experimented with the deployment of PEDs for the treatment of small aneurysms involving the distal circulation of the middle cerebral artery and anterior cerebral artery, as well as in the posterior circulation with promising results [3]. Other drawbacks include the risk of hemorrhagic complications with the possibility of substantial bleeds from giant aneurysms, as well as inadequate thrombosis prevention with antiplatelets that may result in thromboembolic events, as observed in our second patient case. Technical expertise is further contributory to rates of procedural complication as demonstrated in previous studies in which the deployment of a single PED compared to multiple resulted in lower rates of adverse events and complications [3].

Two clinical trials and one large multicenter retrospective analysis exist to date examining the use of PEDs in the treatment of large and giant cerebral aneurysms: the Pipeline for Uncoilable or Failed Aneurysms (PUFS) trial and the Surpass IntraCranial Aneurysm Embolization System Pivotal Trial (SCENT) trial, highlighted in Table 1, and the Korea multicenter trial [8-10].

**Table 2:** Two clinical trials, PUFS (completed) and SCENT (ongoing), analyzing the utility of PED in the treatment of giant and large intracranial aneurysms with final enrollment count, primary outcome follow-up timeframes, primary outcome results, and serious adverse events

Clinical Trial	Clinical Trial Identifier	Enrollment	Primary Outcome	Timeframe	Primary Outcome Results	Serious Adverse Events
Pipeline for Uncoilable or Failed Aneurysms (PUFS)	NCT00777088	108	The number of participants with the occurrence of major ipsilateral stroke or neurological death and occurrence of ipsilateral stroke or neurological death. Count of intracranial aneurysms (IA) evaluated as completely occluded (100%) without major parent artery stenosis.	180 days	73.6% fully occluded intracranial aneurysms	51.4%
Surpass Intracranial Aneurysm Embolization System Pivotal Trial to Treat Large or Giant Wide-Neck Aneurysms (SCENT Trial)	NCT01716117	213	Percent of subjects with 100% occlusion of the aneurysm without clinically significant stenosis (defined as less than or equal to 50% stenosis) of the parent artery based on core laboratory evaluation of the 12-month follow-up angiogram and without any subsequent treatment of the target aneurysm at the 12-month follow-up visit. Subjects experiencing neurological death or major ipsilateral stroke through 12 months.	12 months	62.8% primary effectiveness endpoint composite success, 10.6% primary safety endpoint failure	33.3%

The PUFS trial examined 108 patients in total, with 57% of the patients presenting with aneurysms of the cavernous segment, 25% in supraclinoid, and 27% in the paraophthalmic segment. Among the 39 patients with deficits in presentation, 25 (64%) demonstrated improvement after stenting, with 21 of these patients having complete aneurysm occlusion at six months postoperatively. In total, 80 patients (82%) with aneurysms treated in the PUFS trial and evaluated at six months showed occlusion of the aneurysm, 8% with a neck remnant, 8% with a residual aneurysm, and 2% with indeterminate changes [8]. In Korea, a retrospective review of multiple center data examined the use of the Pipeline stent in unruptured large and giant or fusiform aneurysms. Forty-five patients were found in this report, and the results demonstrated that of the 18 patients with cranial nerve deficits, 15 patients showed improvement post-Pipeline. All patients had good outcomes with mRS of 0 or 1. In 75.9% of patients, there was complete or near-complete occlusion of the aneurysm and decreased sac size in 24.1% of cases. Aneurysms disappeared in 14.3% of cases, decreased in size in 68.6%, and did not change in 17.1% [9]. In the SCENT trial, the initial published reports examined 20 patients enrolled to treat large or giant wide-neck ICA aneurysms, which has since expanded to 213 patients. The majority (60%) of aneurysms in these initial patients were in anterior circulation in the ophthalmic ICA with successful deployment in 19 (95%) of all cases and balloon angioplasty in 42% of cases. There was complete aneurysm neck coverage achieved in all 19 cases. However, the SCENT trial is currently ongoing in completing follow-up assessments on patients enrolled in the study [10].

## Conclusions

Clearly, there is emerging evidence on the utility of PEDs for the treatment of giant cerebral aneurysms. Here, we find success in symptom resolution of our patients within a short period of time indicating reduction of aneurysm mass effect on cranial nerve neuropathy and radiographic evidence in two of our patients at follow-ups demonstrating patency of the stents with aneurysm interval size reduction. While the efficacy of PED placement and symptom resolution reported in current literature and in our patients reported here are promising, more data will be needed on the recurrence rates of giant aneurysms treated with PEDs.

## References

[1] Briganti F, Napoli M, Leone G (2014). Treatment of intracranial aneurysms by flow diverter devices: long-term results from a single center. Eur J Radiol.

[2] Hanel RA, Kallmes DF, Lopes DK (2020). Prospective study on embolization of intracranial aneurysms with the pipeline device: the PREMIER study 1 year results. J Neurointerv Surg.

[3] Patel PD, Chalouhi N, Atallah E (2017). Off-label uses of the Pipeline embolization device: a review of the literature. Neurosurg Focus.

[4] Carneiro A, Rane N, Küker W, Cellerini M, Corkill R, Byrne JV (2014). Volume changes of extremely large and giant intracranial aneurysms after treatment with flow diverter stents. Neuroradiology.

[5] Nossek E, Zumofen D, Nelson E (2015). Use of Pipeline embolization devices for treatment of a direct carotid-cavernous fistula. Acta Neurochir (Wien).

[6] Oishi H, Teranishi K, Nonaka S, Yamamoto M, Arai H (2016). Symptomatic very delayed parent artery occlusion after flow diversion stent embolization. Neurol Med Chir (Tokyo).

[7] Tan LA, Sandler V, Todorova-Koteva K, Levine L, Lopes DK, Moftakhar R (2015). Recovery of pituitary function following treatment of an unruptured giant cavernous carotid aneurysm using Surpass flow-diverting stents. J Neurointerv Surg.

[8] Sahlein DH, Fouladvand M, Becske T (2015). Neuroophthalmological outcomes associated with use of the Pipeline embolization device: analysis of the PUFS trial results. J Neurosurg.

[9] Kim BM, Shin YS, Baik MW (2016). Pipeline embolization device for large/giant or fusiform aneurysms: an initial multi-center experience in Korea. Neurointervention.

[10] Colby GP, Lin LM, Caplan JM (2016). Flow diversion of large internal carotid artery aneurysms with the surpass device: impressions and technical nuance from the initial North American experience. J Neurointerv Surg.

